# Case Report: A case study on the relationship between obesity and estrus in female captive panda

**DOI:** 10.3389/fvets.2025.1552754

**Published:** 2025-04-24

**Authors:** Qiang Zhou, Bo Luo, Bo Yang, Desheng Li, Rongping Wei, Chunyu Xie, Jinhua Yu, Xiangqian Meng, Jianbin Cheng, Ming He, Liu Yang

**Affiliations:** ^1^Key Laboratory of State Forestry and Grassland Administration on Conservation Biology of Rare Animals in the Giant Panda National Park, China Conservation and Research Center for the Giant Panda, Chengdu, China; ^2^Shanghai Wild Animal Park, Shanghai, China; ^3^Chengdu Xinan Gynecology Hospital, Chengdu, China; ^4^Sichuan Jinxin Xinan Women’s & Children’s Hospital (Bisheng), Chengdu, China

**Keywords:** giant panda, abnormal estrus, obesity, hormone levels, reproductive behavior

## Abstract

This study investigated a case of abnormal estrus in a captive adult female giant panda (*Ailuropoda melanoleuca*) (X#, 130 kg) in 2024 to explore the possible relationship between overweight or obesity and estrus behavior in female giant pandas. Behavioral observations and urinary estrogen conjugate/creatinine (EC/CR) measurements revealed a significantly attenuated hormonal profile (peak EC/CR: 34.1 ng/mg vs. normal range 80–150 ng/mg) alongside muted receptivity (e.g., absent tail-lifting). Comparative analysis with historical cases of abnormal estrus (panda 2#: obesity-linked low EC/CR; panda 1#: normal EC/CR with behavioral anomalies) implicated obesity as a driver of reproductive impairment. And several actionable interventions were proposed: bamboo-based dietary reform, structured exercise protocols, and gut microbiota monitoring. This case underscores obesity as a modifiable risk factor in captive panda reproduction, urging integration of metabolic health into breeding management.

## 1 Introduction

The giant panda, a flagship species for biodiversity conservation, requires targeted reproductive research to maintain population health. Their staple diet of bamboo leaves and shoots is rich in cellulose, hemicellulose, carbohydrates, and protein, but contains minimal fat (<4%) [1]. Since bamboo leaves and bamboo shoots are low-quality foods, fat storage is particularly important for pandas, especially during physiological stages such as estrus, pregnancy, and migration [2]. And because the giant panda has a short intestine, the food stays in the intestine for a short time. This digestive system allows pandas to consume a large amount of food, but only a small part of it is converted into energy, and this physiological process makes pandas more prone to obesity [3]. Studies have shown that obesity can lead to endocrine disorders, which in turn affect hormone levels and reproductive behavior, especially during estrus [4–7].

Normal estrus of female giant pandas is one of the key factors for reproduction success. Abnormal estrus may lead to reproduction failure, which affect the sustainable development of the population. Emerging evidences suggests adiposity disrupts hypothalamic–pituitary-ovarian axis signaling in mammals [8–11], yet mechanisms in pandas remain poorly understood. To explore the relationship between obesity and abnormal estrus in captive female giant pandas is of great importance for formulating effective management and conservation strategies. The female giant panda X# was born in October 2017, and her weight has fluctuated around 130 kg since she became an adult. Through the actual investigation of the real estrus behavior and EC/CR levels of panda X#, this study explored the possible relationship between obesity and estrus, and provided practical cases and scientific basis for the management of captive giant pandas.

## 2 Methods

### 2.1 Behavior observation

Reproductive behaviors of X# were documented daily using standardized ethograms for captive giant pandas [12, 13] from the onset of pre-estrus (March 24, 2024) until April 6, 2024 (09:00–17:00), focusing on:

Vulval Morphology: Level of Swelling, color change (pink-white or damp red), and relaxation level of the vulval opening.

Receptivity Testing: To assess receptivity, a standardized tail-lifting test was performed: gentle pressure was applied to the tail region using a blunt probe, and tail position (lifted or not) was recorded as per captive panda estrus evaluation protocols.

Locomotion: Total minutes spent walking, climbing, or exploring.

Rest: Total minutes spent lying or sitting motionless.

Taking Food: Minutes spent consuming bamboo or supplemental feed.

Vocalizations: Frequency of sheep-like bleats.

Scent-marking: Frequency of anogenital rubbing on substrates.

Water-play: Frequency of splashing or submerging in water.

Masturbation: Frequency of stereotypic perineal contact (forelimb gripping, hindlimb flexion).

### 2.2 Hormone level examination

Urine samples were collected at 14 timepoints between March 30 and April 6, 2024. Estrogen conjugate (EC) and creatinine (CR) concentrations were quantified using enzyme-linked immunosorbent assay (ELISA), and the EC/CR ratio was subsequently calculated.

#### 2.2.1 EC detecting assays

Goat Anti-Rabbit IgG (GARG) Preparation: The working GARG solution was prepared at a 1:220 dilution in coating buffer and gently mixed.

Plate Coating: A 200 μL aliquot of the GARG solution was dispensed into each well, sealed, and incubated overnight at 4°C.

Plate Washing/Blocking: After equilibrating TRIZMA buffer to room temperature (RT), the plate was washed three times with wash solution. Each well was then filled with 200 μL of TRIZMA buffer, sealed, and incubated at RT for 1 h.

Sample/Control Preparation: Urine samples were diluted in TRIZMA buffer to appropriate concentrations. High and low controls were equilibrated to RT prior to use.

EC HRP (Horse Radish Peroxidase) and Antibody Preparation: EC HRP and antibody working solutions were prepared at dilutions of 1:100,000 and 1:300,000, respectively, using TRIZMA buffer.

Plate Loading: After discarding the blocking buffer, 150 μL of blank solution (“0”) was added to designated wells. Standards, controls, and samples (50 μL each) were pipetted into assigned wells, followed by 100 μL of EC HRP and antibody solutions. The plate was sealed and incubated at RT for 2 h.

Post-Incubation Washing: Following incubation, the plate contents were discarded, and wells were washed three times with wash solution. Residual liquid was removed by blotting.

TMB (3,3′,5,5′-Tetramethylbenzidine) Substrate Solution/Plate Development: A TMB substrate solution was prepared by dissolving two Sigma T3405 tablets in 20 mL of substrate buffer. Each well received 200 μL of the substrate solution, and the plate was incubated in the dark for 45 min. The reaction was terminated with 50 μL of 4 M H₂SO₄.

Absorbance Measurement: Absorbance was measured at 450 nm using a BioTek Gen5 microplate reader. Blank well optical density (OD) values were maintained between 0.5 and 1.0.

#### 2.2.2 Creatinine detecting assays

Sample Preparation: Urine samples were diluted (1:5–1:100) in dilution buffer.

Plate Loading: Standards, controls, and diluted samples (50 μL each) were loaded in duplicate. Each well subsequently received 50 μL of dH₂O, 50 μL of 0.75 M NaOH, and 50 μL of 0.4 M picric acid. The plate was gently tapped to mix and incubated at RT for 30 min.

Absorbance Measurement: Absorbance was recorded at 490 nm using the BioTek Gen5 system.

### 2.3 Male fertility validation

The male panda involved in mating attempts has a confirmed reproductive history, including successful cub production in prior years. And his behavioral competence (mounting, intromission) aligns with fertile individuals described in captive breeding programs.

## 3 Results

### 3.1 Observation of reproductive behavior

From March 24, X# entered proestrus, with gradual behavioral changes: locomotion increased from 44 min (March 24) to 282 min (April 3), scent-marking rose from 1 to 18 events/day (April 4), and water-play peaked at 18 events on April 4. Stereotypic masturbation emerged on April 4 (1 event) and recurred daily until April 6, coinciding with escalating vocalizations (32 to 65 events/day). On April 6, despite continued masturbation and vocalizations, X# rejected mating. Specifically, it avoided proximity to the male and failed to assume the species-typical dumb standing posture. During the tail-lifting test, X# maintained a dropped tail position when pressure was applied, indicating non-receptivity (Table 1).

**Table 1:** Daily behavioral metrics of X# during 2024 estrus cycle (March 24–April 6).

Date	Locomotion (minutes)	Rest (minutes)	Taking Food (minutes)	Vocalizations (frequency)	Scent-marking (frequency)	Water-play (frequency)	Masturbation (frequency)	Tail-lifting test (lifted/not)
3/24	44	222	214	0	1	1	0	0
3/25	53	257	170	0	2	1	0	0
3/26	85	195	200	0	3	5	0	0
3/27	132	191	157	0	10	5	0	0
3/28	237	109	134	0	9	3	0	0
3/29	230	120	130	0	6	5	0	0
3/30	210	84	186	0	6	3	0	0
3/31	225	168	87	4	5	5	0	0
4/1	237	155	88	6	8	5	0	0
4/2	226	168	86	18	10	7	0	0
4/3	282	146	52	20	12	12	0	0
4/4	274	168	38	32	18	18	1	0
4/5	218	232	30	40	5	4	1	0
4/6	203	178	99	65	3	2	1	0

Vocalizations: Frequency of sheep-like bleats per day. Masturbation: Frequency of stereotypic perineal contact per day. Tail-lifting test: 1 = lifted, 0 = not lifted.

Vulval changes commenced on March 25, characterized by swelling, relaxation of the vulval opening, and color transition from pink-white to damp red.

### 3.2 EC/CR level examination

**Figure 1:**
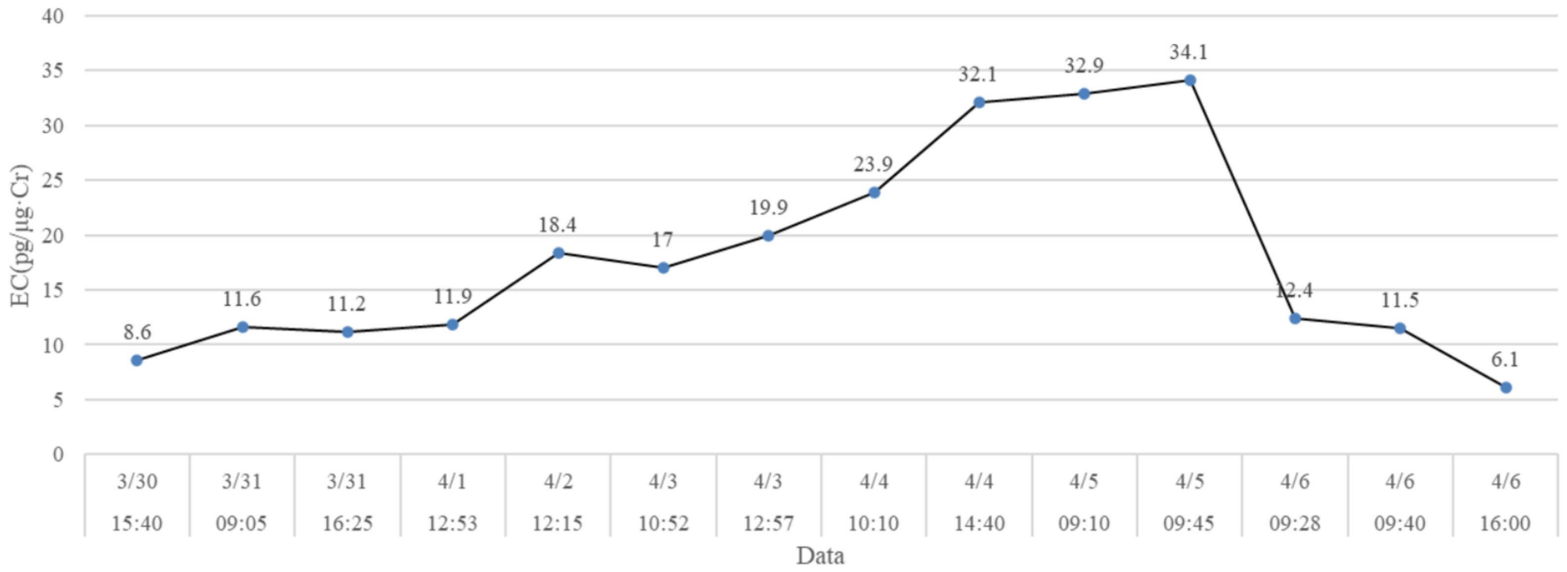
Daily urinary EC/CR levels of X# from March 30 to April 6, 2024. The horizontal coordinate indicates sampling days and times. Values on the dot represents the examined EC/CR.

Urine levels of EC/CR in X# were measured daily between March 30 and April 6, 2024. High-frequency EC/CR measurements revealed a rapid surge from 19.9 ng/mg (April 3 at 10:52) to 34.1 ng/mg (April 5 at 09:45), followed by a sharp decline to 6.1 ng/mg by April 6 at 16:00 (Figure 1). This daily resolution ensured precise tracking of the EC/CR trajectory, with the peak value recorded on April 5 (09:45).

## 4 Discussion

Typically, the EC/CR level in the urine of captive female pandas during peak estrus period is 80–150 ng/mg [14]. However, X# exhibited a peak EC/CR level of 34 ng/mg, significantly below the typical range observed in reproductively normal individuals. Furthermore, while X# displayed typical pre-estrus behaviors (e.g., increased activity, marking) and vocalizations resembling sheep bleats during peak estrus, it failed to exhibit receptive mating postures (e.g., tail-lifting) contrast with normative estrus indicators in captive pandas [12, 13]. Collectively, EC/CR level, behavior and vulvar changes all suggested the X#‘s estrus profile differs from normative patterns in captive adult females, though causation requires further investigation.

Combined with historical data from the same institution, X#‘s atypical estrus (low peak EC/CR, absent receptivity) aligns with a 2019 case involving subadult panda 2# (peak EC/CR: 45 ng/mg, muted estrus behavior) [15]. Both individuals exhibited muted estrus behavior and low peak EC/CR, and notably, both were classified as overweight (2#: 98 kg at 1.5 years old vs. average weight of 1.5 year old subadult as 42.3–53.1 kg [15]; X#: 130 kg vs. adult female reference range 97.34–119.36 kg [16]). Notably, Liao *et al*. identified 110.5 kg as an obesity threshold for adult female pandas, positioning X#’s weight (130 kg) as a potential metabolic risk factor [17].

In contrast, panda 1# [18]—despite atypical mating behavior—displayed normal EC/CR dynamics (peak as 82 ng/mg) and later achieved successful parturition, suggesting a distinct mechanism (e.g., “silent estrus” linked to endocrine dysregulation [19]). The divergence between X#/2# (obesity-driven anomalies) and 1# (behavioral-endocrine mismatch) underscores multifactorial pathways underlying captive panda reproductive challenges.

Cross-species parallels hint at plausible mechanisms: in humans, 50% of polycystic ovary syndrome (PCOS) cases involve overweight/obesity [20], which disrupts folliculogenesis via androgen excess and granulosa cell dysfunction [21]. Similarly, livestock studies document obesity-induced estrus anomalies in sows, ewes, and cows [22–27]. Critically, hypothalamic crosstalk between leptin and estrogen signaling—a central regulator of energy-reproduction balance—may underpin these disruptions. Adipocyte-derived leptin resistance in obesity blunts estrogen’s anorexigenic effects while impairing gonadotropin-releasing hormone (GnRH) pulsatility, thereby decoupling metabolic status from reproductive readiness [28]. Collectively, these lines of evidence position obesity-induced metabolic imbalance as a critical, modifiable risk factor for X#‘s reproductive phenotype. Though direct evidence in pandas is limited, X#‘s attenuated EC/CR levels and leptin-estrogen crosstalk disruptions mirror these mechanisms, strengthening obesity’s plausibility as a contributing factor.

Obesity is a pervasive challenge in zoos, driven by energy-dense diets and sedentary lifestyles [29–37]. Under captive conditions, animals typically have lower activity levels [38]. In gorillas (*Gorilla gorilla*), adiposity correlates with reduced foraging time and elevated insulin resistance [39], while over 70% of captive Asian elephants (*Elephas maximus*) in China exhibit obesity, primarily attributed to insufficient outdoor activity and excessive high-calorie feed provision [40]. In various zoos, there are usually relevant laws and regulations that prohibit the withholding of food from animals [41]. However, to enhance the exhibition effect, keepers may feed the zoo animals excessive amounts of feed. Considering the actual zoo conditions that differ from the wild environment, this study proposes the following methods for comprehensive intervention in the obesity issues of large captive animals in zoo, such as captive giant pandas, including dietary control, behavioral enrichment, and physiological monitoring. Firstly, the obesity of pandas is related to feeding management and public sentiment. The management of panda care should be established on its own scientific feeding methods and not let public sentiment dictate the feeding approach. Secondly, the structure and quantity of the panda’s food should be dynamically adjusted based on individual conditions. Dietary reform should prioritize substituting soybean and egg components in wowotou with bamboo powder – a strategy that reduces crude fat content while maintaining satiety through enhanced fiber intake, mirroring successful interventions in obese primates [42]. Concurrently, high-sugar treats (bamboo shoots, honey, apples) should be restricted of daily intake and reserved for enrichment purposes. It is particularly important to note that during estrus or mucus excretion, when pandas naturally reduce wowotou consumption, caretakers must avoid compensatory high-sugar incentives (e.g., honey-coated feeds) to prevent energy surplus. Thirdly, increase the activity levels of pandas. Environmental enrichment—such as elevated feeders, puzzle devices, and scent trails—can extend foraging time, mimicking wild behaviors. Structured exercise sessions (e.g., guided exploration) further elevate daily energy expenditure, as demonstrated in other obese zoo animals’ trials [African (*Loxodonta africana*) and Asian (*Elephas maximus*) elephants [43], Gorillas (*Gorilla Gorilla Gorilla*) [39], etc. [44]]. To ensure long-term efficacy, gut microbiota composition should be monitored via fecal Gram staining, with *Firmicutes*/*Bacteroidetes* ratios (>1.5) triggering dietary recalibration. This integrated approach recognizes that captivity-induced obesity stems from multifaceted imbalances—requiring equally multidimensional solutions.

This study relied on weight thresholds rather than direct adiposity measurements. Future work should explore non-invasive techniques (e.g., bioelectrical impedance) to quantify body fat in pandas. In addition, the absence of direct biomarkers (e.g., blood glucose) limits our ability to diagnose metabolic syndrome conclusively. Besides, longitudinal tracking of X#‘s subsequent estrus cycles (2025–2026) will clarify whether these anomalies persist or represent transient dysfunction. Nevertheless, the convergence of hormonal, behavioral, and comparative evidence positions obesity as a high-priority risk factor meriting proactive management in captive pandas.

## 5 Conclusion

This case study identifies a correlation between obesity (130 kg) and atypical estrus (low EC/CR, absent receptivity) in a captive female panda. By comparing with other pandas exhibiting abnormal estrous, it is suggested that metabolic imbalance may contribute to reproductive dysfunction, though multifactorial pathways likely coexist. While weight management may offer a pragmatic mitigation strategy, controlled trials are essential to establish causality and optimize protocols.

## Data Availability

The original contributions presented in the study are included in the article/supplementary material, further inquiries can be directed to the corresponding author.
